# Fatal ascending aortic aneurysm in a patient with systemic lupus erythematosus: A case report

**DOI:** 10.1002/ccr3.7696

**Published:** 2023-07-13

**Authors:** Tejash Shahi, Prinska Ghimire, Ujjwal Prakash Khanal, Tulsi Ram Dhakal, Saket Jha

**Affiliations:** ^1^ Institute of Medicine, Tribhuwan University Teaching Hospital Kathmandu Nepal

**Keywords:** aortic aneursym, systemic lupus erythematosus

## Abstract

Aortic aneurysm is a potentially life‐threatening condition with higher incidence in patients with systemic lupus erythematosus(SLE). Patients usually present with nonspecific symptoms and diagnosis is typically made incidentally through imaging studies. Management strategies include medical therapy to control inflammation and hypertension, surgical intervention for large or symptomatic aneursyms, and close monitoring for early detection of complications. We present a case of a 49‐year female with multiple joint pain and other nonspecific symptoms for 7 years. Anti‐ds DNA and ANA titre were significantly high and CT angiogram showed ascending aortic aneurysm measuring 5.5 cm. Conservative management was started with steroids, hydroxychloroquine, and antihypertensives, while awaiting surgery. However she suddenly collapsed, probably due to aneurysm rupture and could not be revived. Our case report therefore emphasizes the importance of close surveillance and timely intervention to minimize the morbidity and mortality in these patients.

## 1 INTRODUCTION

Systemic lupus erythematosus (SLE) is a chronic autoimmune disorder involving multiple organ systems.^1^ With a prevalence of up to 241 per 100,000 people, the disease affects both sexes but has a clear female predominance across all age groups.^2^ Cardiovascular manifestations like pericarditis, valvular heart disease, and endocarditis are common and are usually associated with significant morbidity and mortality.^3^ Aortic aneurysm, however, is not a common feature of SLE, and remains asymptomatic in most patients until detected incidentally on imaging.^4^ Further, aneurysm of the ascending aorta, which can have life‐threatening consequences, is exceedingly rare with only a few cases reported so far.^5^, ^6^, ^7^, ^8^, ^9^

In this report, we present the case of a 49‐year‐old woman with an aneurysm of the ascending aorta in the background of undiagnosed SLE. However, after an episode of sudden, refractory shock, likely due to the aneurysm rupture, she died while awaiting surgical intervention. With this unique case, we aim to alert clinicians to remain vigilant about the potential development of fatal cardiovascular complications like aortic aneurysms in patients with SLE, highlighting the challenges in diagnosis and management in resource‐limited settings.

## 2 CASE PRESENTATION

A 49‐year‐old female presented with recurrent episodes of multiple joint pain and generalized weakness for 7 years, palpitations for two and a half years, and added symptoms of fever, nonproductive cough and shortness of breath for 2 weeks. The pain was initially limited to the small joints of the hands and feet but had progressed to affect the larger joints of both the upper and lower limbs. The pain was severe on waking up in the morning but improved with activity. However, there were several episodes where the pain did not subside with regular activity, and needed the use of over‐the‐counter analgesics. Additionally, she complained of episodic palpitations for two and a half years, which were associated with paroxysms of nocturnal dyspnea along with episodic swelling of lower limbs that would improve with rest. She had visited multiple centers for these complaints and had been treated symptomatically with no diagnosis or further evaluation.

For the past 2 weeks, the patient had been experiencing dry cough and shortness of breath associated with fever reaching up to 100F. Upon further probing, it was discovered that she had increased hair loss, anorexia, and rashes in sun‐exposed regions for the same duration. There was no relevant history of any other medical or surgical intervention in the past, and the family history was found to be insignificant.

On examination, her blood pressure was normal (96/68 mm Hg), but she was tachycardic (112 beats per minute), tachypneic (22 breaths per minute), and hypoxic (SpO_2_ 90% on room air). Pitting edema of both lower limbs, whitish ulceration in the buccal mucosa, nail pittings, ulceration at the tip of the right index finger, and loss of hair and eyebrows were also noted. Chest examination revealed decreased intensity of air entry in bilateral basal auscultatory areas with no added sounds. Precordial examination was normal during initial evaluation at the emergency department. However, a grade I pansystolic murmur was audible upon repeated examinations.

Subsequent investigations showed the following: hemoglobin 9.2 g/dL, packed cell volume (PCV) 28.3, WBC count 4490/mm^3^, platelet counts 152,000, urea 14.9 mmol/L, and creatinine 125 μmol/L. Peripheral blood smear demonstrated pancytopenia with no atypical cells. Her thyroid function test showed an increased TSH level while T3 and T4 levels were within range. Her urine routine examination revealed 2+ albumin, 2–4 WBCs/hpf and no RBCs, and the 24‐h‐urine showed 0.34 g protein. Her anti‐dsDNA titre was >300 IU/mL (>36 considered reactive) and anti‐ANA titre was >500 IU/mL (>48 considered reactive).

**FIGURE 1:**
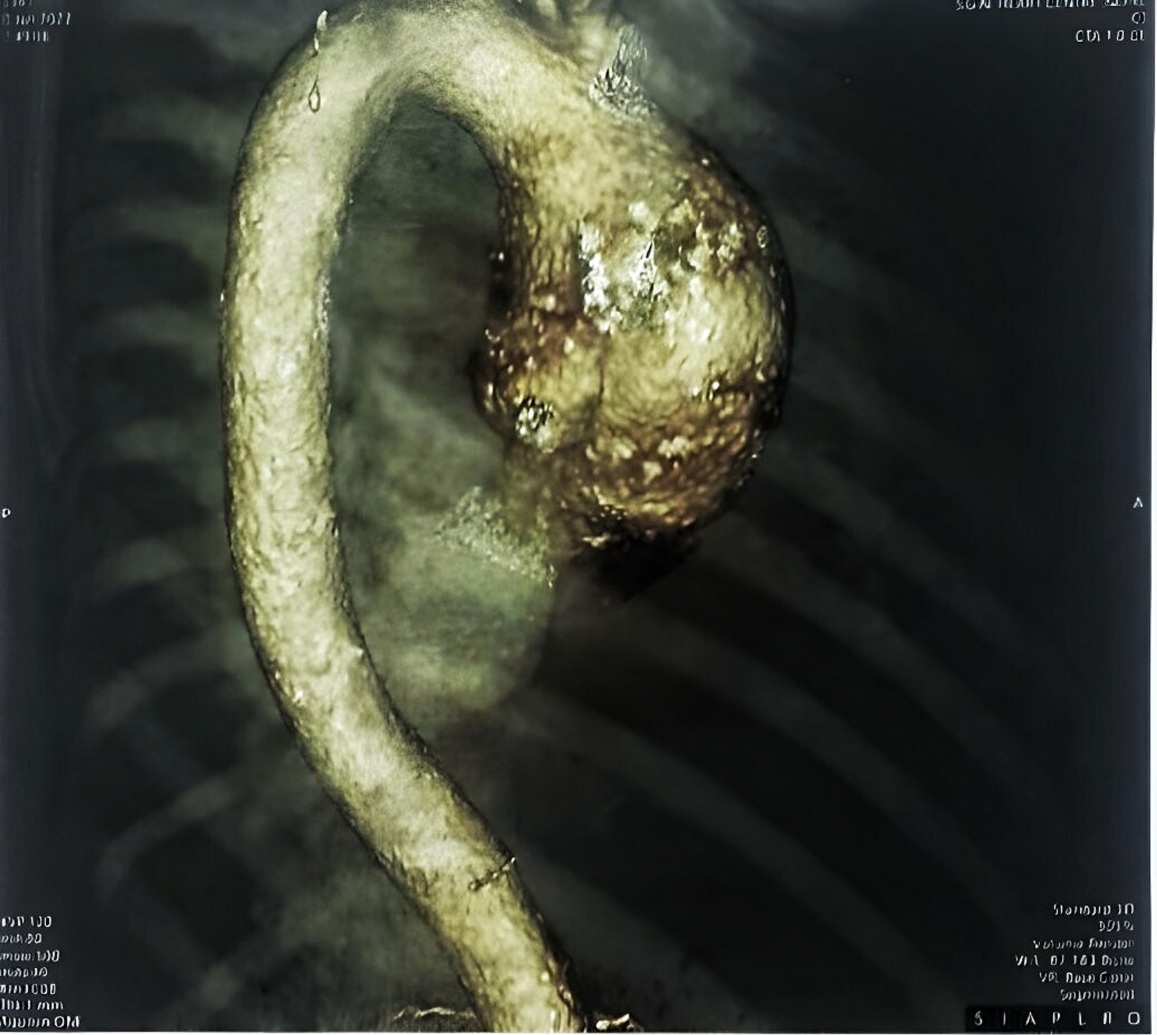
3D reconstruction of the aorta from the CT angiogram of the patient showing an ascending aortic aneurysm.

Echocardiography revealed ascending aortic aneurysm measuring 5.5 cm at its widest diameter, along with moderate aortic regurgitation, severe tricuspid regurgitation, mild mitral regurgitation, moderate pulmonary arterial hypertension, mild pericardial effusion, and no vegetations. CT angiogram demonstrated ectatic ascending aorta with an outpouching of contrast measuring 28 × 21 mm along with diffuse atherosclerotic changes in the abdominal and thoracic aorta in the form of wall thickening and calcification Figure 1.

Based on these investigations and clinical findings, she was diagnosed to have SLE with aneurysm of the ascending aorta and was started on oral prednisolone, hydroxychloroquine, and antihypertensive drugs. However, surgery could not be scheduled immediately owing to the resource constraints posed by the COVID‐19 lockdown, and she was lost to follow‐up. Two months later, she presented with an acute flare‐up of her symptoms, and her reports had further deranged (hemoglobin 8.9 g/dL, PCV 26, WBC 1629/mm^3^, platelets 79,000/mm^3^, urea 16 mmol/L, and creatinine 236 umol/L). She was readmitted for a modified Bentall procedure and was started on intravenous methylprednisolone, hydroxychloroquine, and sildenafil for hematological and hemodynamic optimization.

However, on the eighth day of admission, she suddenly collapsed, and examination revealed a feeble pulse, an unrecordable blood pressure, and a GCS of 3/15. Immediate cardiopulmonary resuscitation was started with the suspicion of rupture of the ascending aortic aneurysm but she could not be revived. The patient's family did not consent for an autopsy.

## 3 DISCUSSION

Systemic lupus erythematosus (SLE) is a chronic autoimmune disorder of multifactorial etiology. The prevalence has been found to range from 3.2 to 70.4 per 100,000 person years across Asian studies, with peak incidence in females from the 3rd to 7th decades of life.^2^, ^10^ The autoimmune process begins as a result of an interaction between genetic, environmental, immunological, and hormonal factors, which can lead to varying degrees of damage of multiple organ systems in the body, commonly involving skin, kidneys, joints, lungs, and blood.^1^, ^11^

Given the variable clinical as well as laboratory manifestations, SLE can pose a significant diagnostic dilemma as observed in our patient who could not be properly diagnosed despite multiple hospital visits for 7 years. Several diagnostic criteria have been proposed and revised to improve the specificity and sensitivity, the most recent one being the 2019 European League Against Rheumatism (EULAR)/ American College of Rheumatology (ACR) criteria, which has a sensitivity and specificity of 96.1% and 93.4%, respectively.^12^

Cardiovascular involvement is common in SLE, where pericarditis is the commonest manifestation seen in up to one‐fourth of all patients. Pericardial effusion, however, can be asymptomatic in over 50% of cases and a diagnosis is usually possible only after an echocardiogram.^13^ The myocardium may also be affected, presenting with symptoms of heart failure and arrhythmia. Additionally, it is not uncommon to find Libman‐Sacks endocarditis and valvular dysfunctions in those with SLE. All or any of these can lead to shortness of breath, as was the case in our patient. Due to increased atherosclerosis in SLE, it is also possible for patients to present with coronary artery disease, peripheral vascular disease, and cerebrovascular diseases.^11^ Presentation with aortic aneurysm(AA), however, is rare.

A nationwide study in Taiwan involving 15,209 patients with SLE reported only 20 cases of AA. However, the risk was found to be higher than that in the general population with an incidence rate ratio of 3.34.^14^ Another study involving 5018 SLE patients echoed this finding, reporting AA in 0.6% of the cases with an odds ratio of 4.5.^15^ Two studies collating cases of SLE with AA from 1968 to 2017 suggested that patients with SLE develop AA at a much earlier age than usual (44.5 and 44.6 years, respectively) with a higher incidence in females.^16^, ^17^ This corresponds to our case, who was a female of 49 years.

The pathophysiology of AA in SLE is not fully understood. Studies suggest aortic aneurysms to be frequently correlated with multiple risk factors including atherosclerosis, prolonged steroid use, and vasculitis.^6^ Older age at the time of diagnosis, male sex, SLE duration longer than 3 years, raised ESR, hypertension, CAD, and CHF are other associated risk factors.^14^, ^18^, ^19^ Thoracic aortic aneurysm (TAA), in particular, was found to be correlated with medial cystic degeneration associated with vasculitis, whereas abdominal aortic aneurysm (AAA) was correlated with atherosclerosis and prolonged steroid use. This difference may be related to the different embryological origin of vascular smooth muscle cells; that of the ascending aorta originate from the neural crest cells whereas that of the descending aorta from the paraxial mesoderm.^4^ Moreover, TAA showed a positive relation with dissection, rupture, and mortality and a negative relation with the 10‐year survival in SLE.^16^ Although we could not look for vasculitic changes in our patient owing to the lack of an autopsy, it is likely that the first mechanism was in play, as suggested by the location and the lack steroid use at diagnosis.

Most patients with uncomplicated aneurysm of the ascending aorta do not exhibit clinical symptoms and are usually detected incidentally on imaging. They can instead present with features of acute (and often fatal) complications, mainly aortic dissection, and rupture.^4^, ^20^ Our patient, however, had complaints of palpitations along with other cardiac symptoms, which prompted an echocardiogram leading to the diagnosis of aneurysm of the ascending aorta.

At present, there are no clinical guidelines for the treatment of AA in SLE specifically. Conservative management usually includes blood pressure control, lipid level optimization, and cessation of smoking. Pulmonary arterial hypertension, if concurrently present, is often treated with PDE5 inhibitors like Sildenafil. In our patient, too, it was preferred considering its established safety, efficacy, and easy availability. However, there is emerging evidence to suggest sildenafil might aggravate elastin degradation, thereby accelerating chances of rupture, and hence requiring caution before use.^21^

The decision to pursue surgery is often guided by the size of aneurysm since it is directly related to the risk of complications.^22^ The normal mean diameter of the ascending aorta is 2.86 cm in both sexes.^23^ The annual risk of dissection or rupture for TAA is around 2% for <5 cm diameter, 3% for 5–5.9 cm, and 7% for >6 cm.^24^ Furthermore, it has been shown that the rate of expansion of TAA, not specific to any etiology, increases to 0.79 cm per year for diameter >5 cm compared to 0.17 cm per year for those <5 cm.^25^ The Canadian society for vascular surgery thus recommends surgery when the diameter of the aneurysm is greater than 5.5 cm in males and 5.0 cm in females.^26^ Additionally, symptomatic TAAs with pain or any complication are also recommended for immediate surgical repair.^4^ Compared to fusiform aneurysms, saccular aneurysms are more prone to rupture, and therefore require more aggressive management.^27^ Since the diameter of the ascending aorta was 5.5 cm in our patient and it had an additional saccular component, she was eligible for surgery.

There are minimally invasive surgical options for the treatment for aneurysms in other parts of the aorta, but the options are limited for aneurysms of the ascending aorta, with the primary treatment being either aortic replacement or repair.^23^, ^28^ In case of concurrent aortic valvular disease, combined valvular replacement maybe performed simultaneously as well.^29^ However, surgery is often associated with multiple adverse outcomes, as demonstrated by a 2000 study of 443 patients where up to 11.5% of the patients either died or had permanent neurological injury.^30^ Another 2010 study echoed similar findings, reporting perioperative mortality of 3.7% in high volume centers and 8.3% in low volume centers.^31^ Considering these risks and the active flare‐up of rheumatological symptoms at presentation in our patient, surgery could not be performed immediately and was planned only after an inpatient optimization before she died of aneurysm rupture.

## 4 CONCLUSION

Aneurysm of the aorta is a rare but life‐threatening complication of SLE. It is important for clinicians to be aware of this complication, to remain vigilant for the early symptoms and to plan possible steps of action so as to prevent adverse outcomes resulting from diagnostic or therapeutic delays. Moreover, considering the lack of reliable clinical indicators and the potentially fatal nature of AAs, the place for routine screening for AA in patients with SLE needs to be further explored.

## AUTHOR CONTRIBUTIONS

**Tejash Shahi:** Writing – original draft; writing – review and editing. **Prinska Ghimire:** Conceptualization; data curation; writing – original draft; writing – review and editing. **Ujjwal Prakash Khanal:** Conceptualization; writing – original draft; writing – review and editing. **Tulsi Ram Dhakal:** Data curation; writing – original draft. **Saket Jha:** Conceptualization; supervision.

## FUNDING INFORMATION

No funding was required in the preparation of this case report.

## CONFLICT OF INTEREST STATEMENT

None of the authors have any conflicts of interest to disclose.

## ETHICS STATEMENT

This study is in compliance with the declaration of Helsinki.

## CONSENT

Written informed consent was obtained from the patient for publication of this case report in accordance with the journal's patient consent policy.

## Data Availability

All data generated or analyzed during this study are included in this published article.
